# Performance of a Flexible Versus Rigid Fetoscope in the Simulated Management of Twin‐to‐Twin Transfusion Syndrome

**DOI:** 10.1002/pd.70213

**Published:** 2026-07-05

**Authors:** Emmanuel Rault, Jerome Massardier, Elsa Limouzin, Mona Massoud, Cyril Huissoud, Anthony Atallah

**Affiliations:** ^1^ Department of Obstetrics and Fetal Medicine Hospices Civils de Lyon Femme Mère Enfant Hospital Bron France; ^2^ Centre de Compétence Maladies Rares pour Les Complications Liées au Placenta des Grossesses Monochoriales (PaRaDiGM) Bron France; ^3^ Department of Obstetrics and Fetal Medicine Centre Hospitalier Lyon Sud University Hospital Hospices Civils de Lyon Pierre Bénite France; ^4^ Claude Bernard University Lyon 1 Lyon France

## Abstract

**Objective:**

To assess the feasibility and operative performance of a flexible fetoscope (FF) compared with a rigid fetoscope (RF) in the simulated management of twin‐to‐twin transfusion syndrome (TTTS) with anterior placentation.

**Method:**

A randomized, non‐blinded crossover trial was conducted during a national training session for fetal surgeons. Twenty participants each performed a simulated Solomonization procedure using both the FF and RF in a high‐fidelity TTTS simulator. The FF consisted of a single‐use flexible video‐endoscope inserted into a 10 Fr, 230‐mm stainless steel cannula. Outcomes included the visualization of the recipient cord insertion, the completeness of Solomonization, operative time, tangential vessel approach, and spatial analysis of the Solomonization path to quantify equatorial deviation and its direction relative to the intertwin membrane.

**Results:**

No statistically significant differences were observed between the FF and RF in recipient cord insertion visualization (80% vs. 40%, *p* = 0.17), complete Solomonization (95% vs. 70%, *p* = 0.58) and total procedure duration (11.30 ± 3.09 vs. 9.00 ± 2.36 min; *p* = 0.08). The overall vessel approach angle was slightly more perpendicular with the FF (median 3.5[IQR 3.0–4.7]) than with the RF (3.0[2.2–3.7]; *p* = 0.08). Spatial analysis revealed a larger equatorial deviation area with the FF (31.1 ± 10.3 vs. 17.8 ± 7.1 cm^2^; *p* = 0.01) and a more anterior trajectory (signed deviation 15.6 ± 10.2 vs. −15.0 ± 17.0 cm^2^; *p* < 0.01).

**Conclusion:**

Flexible fetoscopy appears feasible in the setting of anterior placentation in simulated TTTS surgery, supporting its potential clinical value and the need for dedicated training before clinical translation.

## Key Summary

- What's already known about this topic?
  ◦ Fetoscopic laser surgery is the standard treatment for twin‐to‐twin transfusion syndrome (TTTS), but anterior placentation hampers access and visualization. Flexible fetoscopes have been developed and evaluated in experimental and preclinical settings, suggesting potential advantages for placental exploration.
- What does this study add?
  ◦ In a randomized simulation‐based study, flexible fetoscopy was feasible and enabled more anterior trajectories and improved visualization of anastomoses compared with a rigid instrument.
  ◦ This study provides a standardized comparative assessment combining quantitative spatial metrics and operator‐reported outcomes, highlighting potential clinical utility and training requirements.

## 1 Introduction

Twin‐to‐twin transfusion syndrome (TTTS) is the most common complication of monochorionic twin pregnancies and is associated with significant fetal morbidity and mortality [1]. To date, the gold standard treatment consists of fetoscopic laser surgery aimed at coagulating placental vascular anastomoses responsible for the syndrome using a rigid fetoscope and a laser fiber [2]. This highly delicate procedure is performed in specialized centers and remains associated with considerable fetal loss, with dual fetal survival reported in only approximately 65% of cases and survival of at least one twin in around 80% [3].

Surgical morbidity may be compromised in cases of anterior placentation, which makes complete exploration of the placental surface more challenging, or even impossible, with the currently available rigid fetoscopes [4]. These instruments do not allow for tip deflection, and access to the placental surface relies solely on its natural curvature, which is often insufficient to enable complete visualization and treatment of all anastomotic zones. As a result, coagulation may sometimes be incomplete, non‐selective, or performed using a tangential approach, thereby increasing the risk of complications. Across multiple surgical fields—including urology, neuroendoscopy, and digestive endoscopy—new flexible‐tip endoscopic devices have been developed, providing greater range of motion and improved access to anatomically complex areas. Inspired by these techniques, flexible fetoscopes have been introduced in fetal surgery, enhancing accessibility to difficult placental regions. Preliminary studies with these new instruments are promising and emphasize the need for continued evaluation [5].

Flexible fetoscopes have not yet been adopted in routine clinical practice, raising two key challenges: evaluating the device's performance in realistic surgical conditions and ensuring adequate training for fetal surgeons. Given the low incidence of TTTS, opportunities for hands‐on experience remain limited, making high‐fidelity simulation particularly valuable for the acquisition and assessment of skills required for rare fetal interventions. Simulation‐based training has already proven effective in improving performance in complex fetal procedures [6, 7, 8].

Therefore, the aim of this study was to evaluate the feasibility of using a FF and to compare its performance with that of a rigid fetoscope in a high‐fidelity simulation model of TTTS with an anterior placental insertion.

## 2 Methods

Participants were recruited during the annual national meeting of French reference centers for rare diseases related to placental complications of monochorionic twin pregnancies held in May 2025. All participants were fetal surgeons with prior experience in rigid fetoscopic laser surgery. Experience levels and specific data on the number of procedures performed by each operator are reported in Table 1.

**TABLE 1:** Baseline characteristics of participants according to randomization group (rigid vs. flexible fetoscope).

Variable	Rigid (*n* = 10)	Flexible (*n* = 10)
	*n* (%)	*n* (%)
Age (years) mean (± SD)	45.9 (± 9.9)	46.3 (± 11.6)
Fetal procedures performed		
Amniocentesis	10 (100)	10 (100)
CVS	10 (100)	10 (100)
IUT	10 (100)	10 (100)
Fetal drains	8 (80)	9 (90)
Cord coagulation	10 (100)	9 (90)
Other surgeries	2 (20)	1 (10)
Years of experience in fetal medicine mean (± SD)	13.9 (± 8.90)	14.1 (± 11.2)
Years of experience performing fetoscopic laser		
< 5	3 (30)	7 (70)
5–10	2 (20)	0
> 10	5 (50)	3 (30)
Number of fetoscopic laser performed as the primary operator/year		
10–25	2 (20)	4 (40)
< 10	8 (80)	6 (60)
Fetoscopic laser in center/year		
10–25	5 (50)	6 (60)
< 10	4 (40)	3 (30)
> 50	1 (10)	1 (10)
Years since center started fetoscopic laser mean (± SD)	14.6 (± 8.25)	12.60 (± 6.77)
Numbers of fetoscopic surgeons in center		
1	1 (10)	1 (10)
2	6 (60)	7 (70)
3	1 (10)	1 (10)
4	1 (10)	0
5	1 (10)	1 (10)

Abbreviations: CVS, chorionic villous sampling; IUT, intrauterine transfusion.

Each participant completed a baseline questionnaire collecting demographic and professional characteristics, including age, types of fetal procedures performed, years of experience in fetal medicine, years of experience performing fetoscopic laser, and the annual number of fetoscopic laser procedures performed as the primary operator. Center‐specific information was also gathered: the annual number of fetoscopic laser procedures performed, the number of years the center had been offering fetoscopy, and the number of fetoscopic surgeons per center.

We conducted a randomized, non‐blinded, crossover trial. Participants were randomized in a 1:1 ratio into two groups using the online tool www.random.org: one group performed the procedure first with the rigid fetoscope (RF) followed by the flexible fetoscope (FF), while the other group used the FF first followed by the RF. Blinding was not feasible due to the nature of the intervention.

**FIGURE 1:**
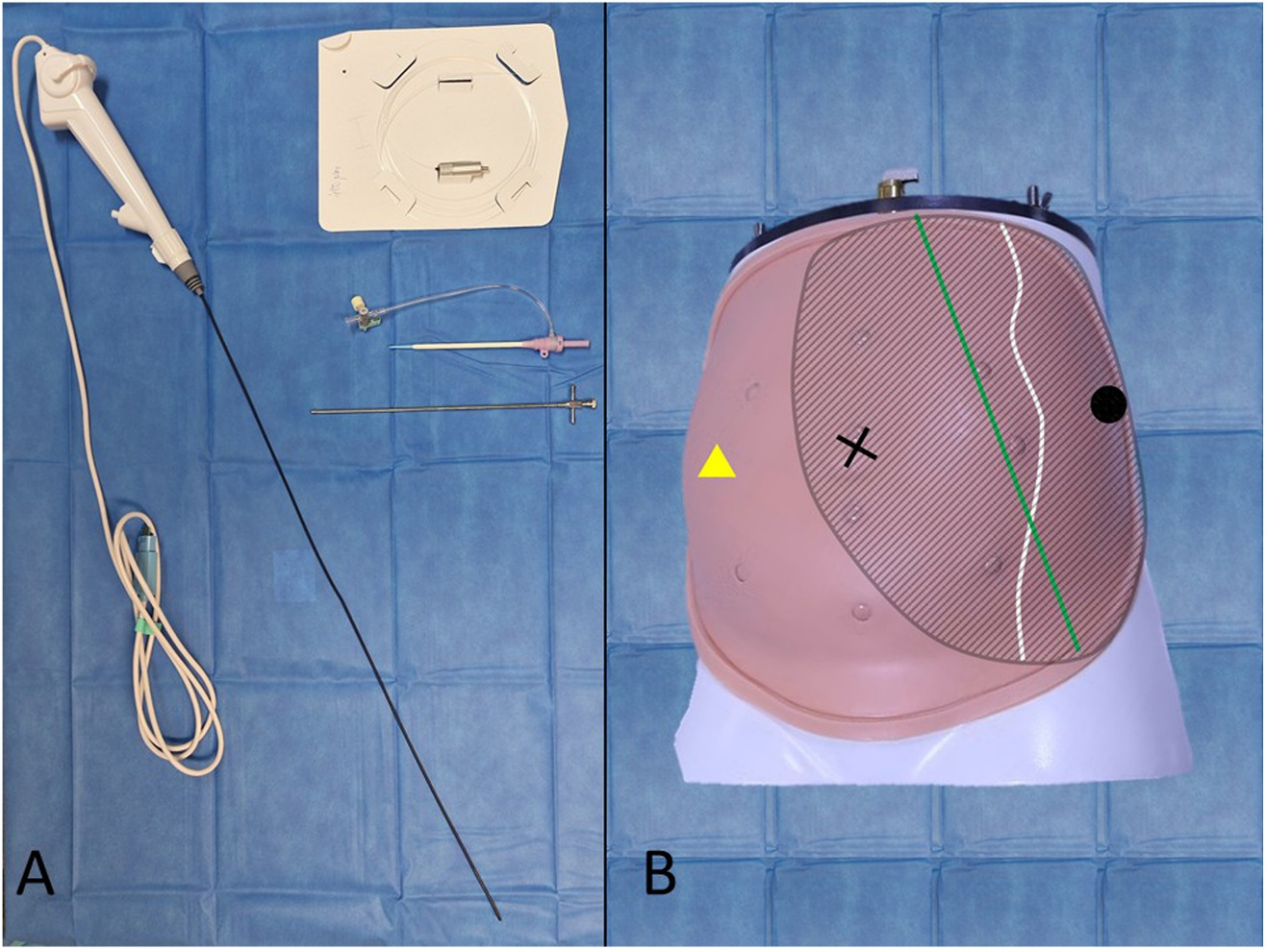
(A) Flexible fetoscope used in the study. A single‐use flexible video‐endoscope was inserted into a 10 Fr, laser fiber and 230‐mm stainless steel cannula for intrauterine guidance and orientation. (B) External view of the simulator showing placental position and cord insertions. The yellow triangle indicates the trocar entry point. The hatched area represents the placental position. The black cross and black circle represent the recipient and donor cord insertions, respectively. The white line represents the placental vascular equator, and the green line represents the insertion of the intertwin membrane.

The FF used was a single‐use flexible video‐endoscope (Temo Medical) with a diameter of 7.5 Fr, a length of 67 cm, and a 3.6 Fr working channel with Luer lock connection. The tip allowed bidirectional deflection (up to 285° upward and downward) and 120° rotational capacity. Deflection capacity varied according to the laser fiber introduced: maximal deflection of approximately 280° without fiber, 180° with a 400‐μm fiber, and 90° with a 600‐μm fiber. The device was inserted into a 10 Fr, 230 mm straight stainless steel cannula for intrauterine guidance and orientation (Supporting Information S1: (video), Figure 1A). The RF used was a straight 2.0‐mm instrument with an operating sheath and integrated working channel (11630AA, Karl Storz, Tuttlingen, Germany), commonly used in clinical practice and introduced through the same 10 Fr cannula.

Simulation was performed using a previously described high‐fidelity fetoscopic simulator (Francis LeBouthillier, Surgical Touch, Toronto, Canada) [9]. The simulator consists of a universal maternal abdomen into which interchangeable modules can be inserted, including those for amniocentesis, chorionic villus sampling, or fetal endotracheal occlusion. For this study, the monochorionic placenta module configured for TTTS was used. The abdominal wall was represented by a silicone interface, with the trocar pre‐positioned in the left flank at the beginning of each procedure. The uterine cavity was filled with fluid to replicate intrauterine conditions. Donor and recipient twins were represented by anatomically realistic fetal models. The placenta was positioned in an anterolateral right location, with anterior cord insertion for the recipient and lateral right insertion for the donor beneath the recipient. The vascular equator ran anterolaterally to the right with anterior insertion of the interamniotic membrane. Two‐thirds of the equatorial course was located beneath the membrane (posterior to the insertion of the intertwin membrane). This configuration ensured potential accessibility of the equator with both RF and FF, while its posterior location relative to the insertion of the intertwin membrane allowed us to evaluate how each instrument influenced the operator's ability to perform coagulation in this context and to achieve complete equatorial Solomonization (Figure 1B).

The study was conducted in three phases. In Phase 1, participants were introduced to the FF and allowed to handle it outside the simulator. In Phase 2, each participant performed a simulated fetoscopic procedure using the device allocated first by randomization. The goal of each procedure was complete Solomonization of the placental vascular equator [10]. Operators initially explored the placenta without the laser fiber. When coagulation was deemed necessary, a laser fiber was inserted into the working channel and activated using a foot pedal that reproduced the characteristic laser activation sound. The Solomonization path was thus defined as the trajectory traced by the operator using auditory foot‐pedal cues, without actual delivery of laser energy, in order to preserve the simulator. All procedures were performed individually without an assistant. If required, minimal assistance was allowed solely for trocar stabilization; otherwise, the task was conducted as a two‐hand procedure. During Phase 2 only, two observers (A.A. and E.R.) jointly annotated each participant's Solomonization path in real time on a standardized template, producing a single consensual trajectory for each procedure. These recorded trajectories were subsequently used for post‐procedure analysis. The standardized placental template was not available to participants during the procedure and was used exclusively for post‐procedure annotation. Operators were not provided with a predefined target trajectory and determined the Solomonization path based on their own visual assessment of the simulated placenta. The primary outcome was feasibility, defined as the visualization of the recipient cord insertion, the visualization of the intertwin membrane and the completeness of Solomonization. Operator performance was assessed based on the operative time, the occurrence of at least one tangential approach to a vessel and the overall perpendicularity of the vessel approach using a 0–5 Likert‐type scale (0 = always tangential, 5 = always perpendicular). The assessment of vessel approach was based on visual evaluation by the observers. In Phase 3, participants repeated the procedure using the second device to allow direct within‐subject comparison; however, the Solomonization path was not evaluated.

**FIGURE 2:**
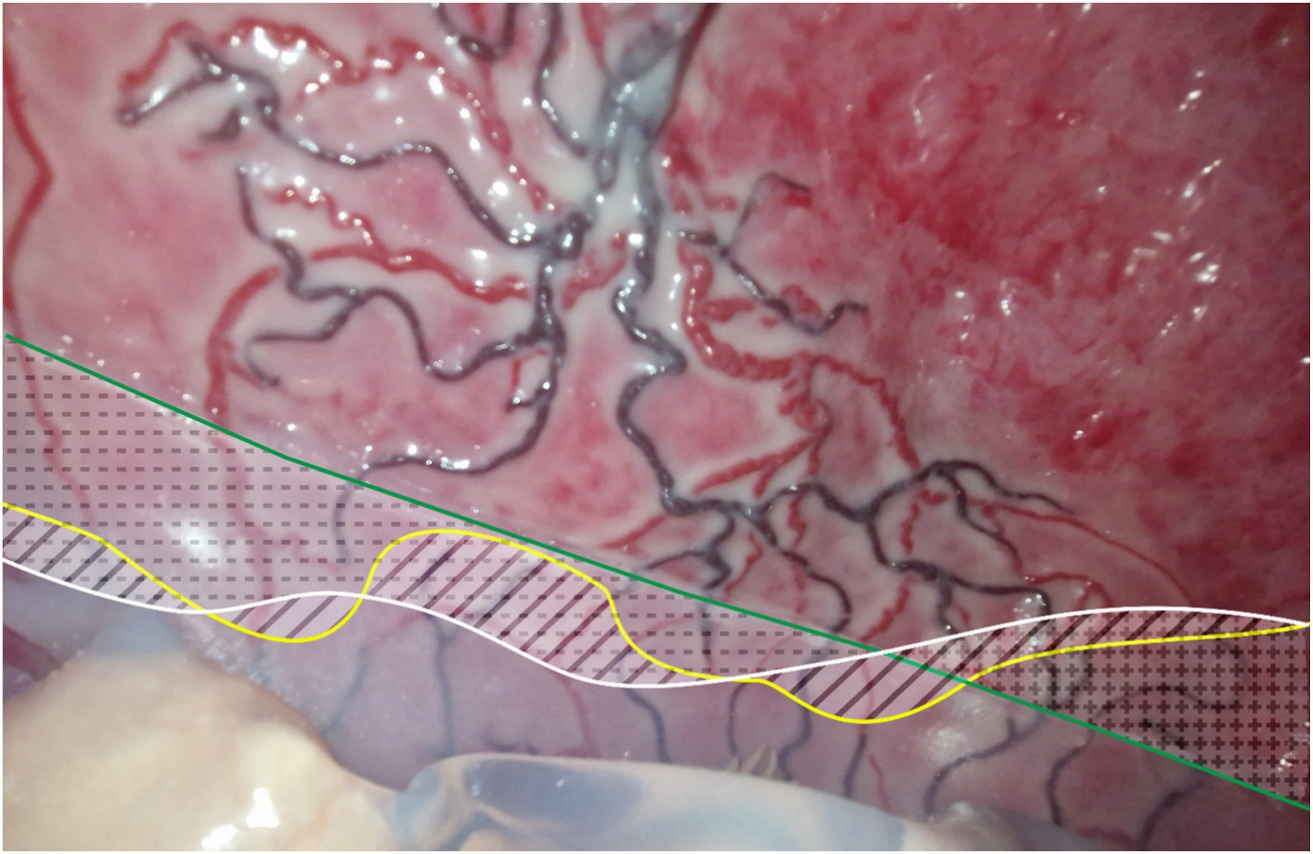
Placental template illustrating the definition of spatial analysis variables. The yellow curve represents the theoretical vascular equator, the green curve represents the insertion of the interamniotic membrane, and the white line is an example of a solomonization path. The hatched area corresponds to the equatorial deviation area, while the zone marked with “+” and “−” symbols represents the signed deviation area: “+” indicates regions located anterior to the insertion of the membrane, and “−” indicates regions located posterior to it.

To further assess the operative performance, we performed a post‐procedure spatial analysis of the Solomonization paths. For each participant who achieved a complete Solomonization, the trajectory was manually outlined on an individual standardized placental template, and a curve was drawn to trace the path performed with each fetoscope. The area enclosed between the Solomonization trajectory and the theoretical vascular equator, expressed in square centimeters (cm^2^), was used as a marker of deviation from the optimal coagulation plane and defined as the equatorial deviation area. In addition, we computed a signed deviation area to assess the directionality of the coagulation path relative to the membrane. The area enclosed between the Solomonization trajectory and the membrane was calculated, with values located anterior to the insertion of the intertwin membrane considered positive, and those located posterior to it considered negative. This variable, expressed in cm^2^, provided a composite measure of both the magnitude and anatomical direction of deviation. The placental template with the definition of the different areas used is presented in Figure 2. We also measured the length of the Solomonization path located beneath the membrane, expressed in centimeters, and defined this as the coagulation length beneath the membrane. Finally, we recorded the number of operators who coagulated beneath the membrane in cases where the vascular equator was located above it.

Participants filled out a post‐simulation questionnaire using Likert scales (1 = strongly disagree to 5 = strongly agree) after completing both procedures. The questionnaire assessed their perceptions of the FF compared to the RF, including its usefulness for identifying vascular anastomoses, perceived quality of operative performance, ergonomics, and sense of control as well as its suitability for anterior placental insertions. Participants also evaluated the ease of handling the FF and the perceived need for additional training prior to clinical use. Their views on the potential integration of the FF in their own center were explored along with the likelihood of its acquisition within the coming year. Finally, participants assessed the realism and educational value of the simulator, including its anatomical and technical fidelity, its relevance for evaluating new operative devices, and its usefulness for initial fetoscopy training.

Continuous variables were summarized as mean (standard deviation) or median (interquartile range) depending on their distribution. Normality was assessed using the Shapiro–Wilk test. Paired comparisons between procedures performed with the RF versus FF were conducted using the paired Student's t‐test for normally distributed variables and the Wilcoxon signed‐rank test for non‐normally distributed variables. Categorical variables were compared using the Chi‐squared test, or the Fisher's exact test, as appropriate. Mean Solomonization paths were derived by averaging polynomial fits of individual paths within each group. All statistical tests were two‐tailed, and a *p*‐value < 0.05 was considered statistically significant. Statistical analyses were performed using R: A Language and Environment for Statistical Computing (version 4.2.2). ImageJ (National Institutes of Health, Bethesda, MD, USA) was used for all image‐based measurements.

All participants provided written informed consent prior to inclusion. Participation was voluntary, and involvement in the study had no impact on professional relationships or clinical responsibilities. The study was conducted in accordance with the ethical principles of the Declaration of Helsinki and was approved by the Institutional Review Board (approval number CEROG 2025‐OBS‐0902) [11].

## 3 Results

A total of 20 fetal medicine specialists participated in the study. Mean age was 45.9 years (± 9.9) in the RF group and 46.3 years (± 11.6) in the FF group. All participants reported prior experience with invasive fetal procedures, including amniocentesis, chorionic villus sampling, intrauterine transfusion, and fetoscopic laser surgery (Table 1).

Procedural performance outcomes are presented in Table 2. The visualization of recipient cord insertion was more frequent with the FF than with the RF (80% vs. 40%, *p* = 0.17). Visualization of the intertwin membrane was achieved in all cases with the FF and in 90% with the RF. Incomplete Solomonization occurred in 3 participants using the RF and 1 using the FF (*p* = 0.58). The duration of the procedure prior to fetoscope insertion was significantly longer with the FF than with the RF (3.40 ± 1.17 vs. 2.10 ± 0.74 min, *p* = 0.01). No significant differences were observed in post‐insertion duration (7.90 ± 2.92 vs. 6.90 ± 1.91 min, *p* = 0.38) or in total procedure duration (11.30 ± 3.09 vs. 9.00 ± 2.36 min, *p* = 0.08). The proportion of participants who approached at least one vessel tangentially was lower with the FF than with the RF (20% vs. 70%, *p* = 0.07). The overall approach angle, rated on a 0–5 Likert‐type scale, was slightly more perpendicular with the FF (median 3.5 (IQR 3.0–4.7)) than with the RF (3.0 (2.2–3.7), *p* = 0.08).

**TABLE 2:** Comparison of visualization, procedural timing, and operative performance between rigid and flexible fetoscope groups.

Operative performance	Rigid (*n* = 10)	Flexible (*n* = 10)	*p*
	*n* (%)	*n* (%)	
Recipient cord insertion visualized	4 (40)	8 (80)	0.17
Intertwin membrane visualized	9 (90.0)	10 (100.0)	1.00
Procedure duration before insertion, mean (± SD) (min)	2.10 (± 0.74)	3.40 (± 1.17)	< 0.01
Procedure duration after insertion, mean (± SD) (min)	6.90 (± 1.91)	7.90 (± 2.92)	0.38
Total procedure duration, mean (± SD) (min)	9.00 (± 2.36)	11.30 (± 3.09)	0.08
At least one vessel approached tangentially	7 (70)	2 (20)	0.07
Perpendicular approach	3.0 (2.2, 3.7)	3.5 (3.0, 4.7)	0.08
Incomplete solomonization	3 (30)	1 (10)	0.58

	**Rigid (*n* = 7)**	**Flexible (*n* = 9)**	
Equatorial deviation area, mean (± SD) (cm^2^)	17.8 (± 7.1)	31.1 (± 10.3)	< 0.01
Coagulation length beneath the membrane, (median [IQR]) (cm)	15.2 [12.3, 18.0]	0.0 [0.0, 3.7]	< 0.01
Signed deviation area relative to membrane, mean (± SD) (cm^2^)	−15.0 (± 17.0)	15.6 (± 10.2)	< 0.01

**FIGURE 3:**
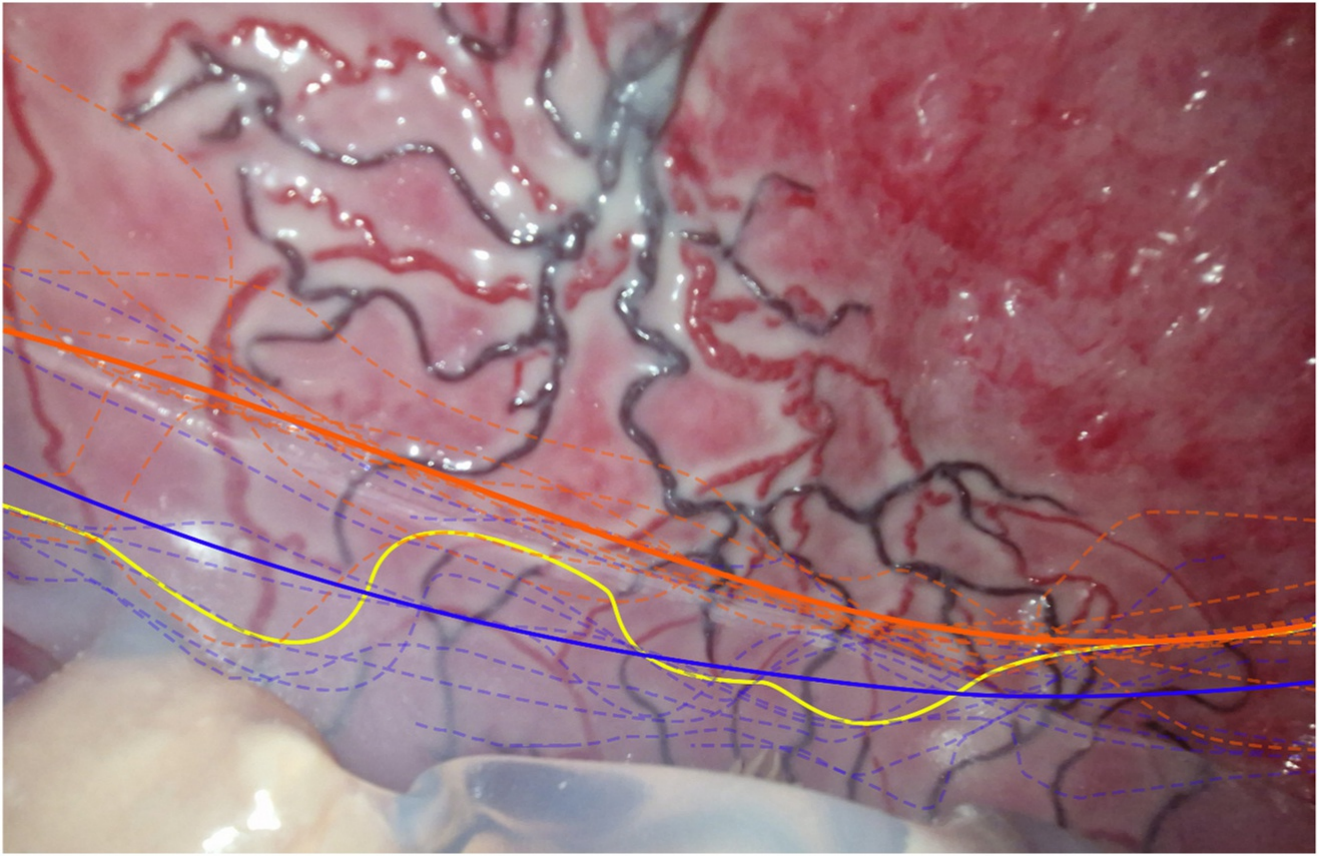
Annotated solomonization paths performed by all participants and means solomonization paths for each group using the rigid and flexible fetoscopes. The yellow line represents the placental equator. Orange lines represent procedures performed with the flexible fetoscope, and blue lines represent procedures performed using the rigid fetoscope. Dotted lines indicate individual solomonization paths, while solid lines indicate the mean solomonization path.

Spatial analysis of the Solomonization paths demonstrated a significantly larger equatorial deviation area with the FF than with the RF (31.1 ± 10.3 cm^2^ vs. 17.8 ± 7.1 cm^2^; *p* = 0.01). The signed deviation area was significantly different between the two groups, with a negative mean value when using the RF (−14.98 ± 17.05 cm^2^) and a positive mean value when using the FF (15.58 ± 10.19 cm^2^; *p* = 0.001), indicating a more anterior coagulation trajectory below the insertion of the membrane with the RF and a preferential course above the membrane with the FF. Solomonization path was observed beneath the membrane in 9 participants (90%) in the RF group and 4 participants (40%) in the FF group (*p* = 0.06). In two cases, part of the Solomonization path was located beneath the membrane despite the vascular equator being located above it; both events occurred in the RF group. The coagulation length beneath the membrane was significantly lower with the FF (0.0 mm (IQR 0.0–3.7)) than with the RF (15.2 mm (IQR 12.3–18.0); *p* = 0.01). All annotated Solomonization paths and mean Solomonization paths for each group are presented in Figure 3.

Participant feedback is summarized in Table 3. The FF was considered to improve the identification of anastomoses (95% strongly agreed) and to enhance operative performance (80% strongly agreed). Although most participants found the FF more ergonomic than the RF (median Likert score 4 (IQR 3–5)), only 10% found it fully intuitive, and 70% expressed a need for additional training prior to clinical implementation. The FF was considered suitable for anterior placentation, with 70% of participants recommending its use in such cases. Most respondents (90%) believed that the device could be integrated into their own centers, and over half anticipated potential acquisition within the following year. The simulation platform was rated as sufficiently realistic to distinguish performance between instruments (median 4 (IQR 4–5)).

**TABLE 3:** Post‐simulation evaluation of the flexible fetoscope and simulation environment using likert scale responses.

Items	1 (Strongly disagree)	2	3	4	5 (Strongly agree)
Subjective comparison of devices					
The flexible fetoscope facilitated identification of anastomoses				1 (5)	19 (95)
The flexible fetoscope allowed better operative performance				4 (20)	16 (80)
The flexible fetoscope seemed more ergonomic than the rigid fetoscope		1 (5)	5 (25)	8 (40)	6 (30)
I would recommend using the flexible fetoscope in cases of anterior placenta			1 (5)	5 (25)	14 (70)
Ease of use					
The flexible fetoscope was intuitive to use		1 (5)	6 (30)	11 (55)	2 (10)
Additional training would be necessary before clinical use		2 (10)	7 (35)	4 (20)	7 (35)
Adoption potential					
I believe this device could be integrated into my center				2 (10)	18 (90)
My center could acquire this device within the next year	1 (5)	1 (5)	4 (20)	3 (15)	11 (55)
Simulator realism and relevance					
The simulator is realistic enough to differentiate the performance of the devices being tested				12 (60)	8 (40)

## 4 Discussion

In this randomized simulation‐based study, we demonstrated that the use of a FF is feasible for the management of TTTS. On this high‐fidelity model, complete Solomonization was achieved in a greater proportion of cases with the FF compared with the RF. While its use resulted in a more peripheral coagulation trajectory relative to the vascular equator, this path was generally located in a more anterior position and less frequently crossed the intertwin membrane. Participants emphasized the potential clinical value of this novel device and expressed broad support for the implementation of dedicated training before its introduction into practice.

The management of TTTS remains a major challenge for fetal therapy teams, with surgical quality being a key determinant of outcome. Anterior placentation frequently limits full visualization of the chorionic plate, thereby increasing the risk of incomplete or non‐selective laser surgery and compromising fetal prognosis [4]. Several strategies have been proposed to overcome this difficulty, including extreme maternal tilting with lateral uterine entry, the use of specialized fetoscopes with a 30° lens [12], indentation of the placenta by advancing the trocar to orthogonalize the vessels [13], or laparoscopy‐assisted fetoscopy [14]. However, these approaches remain suboptimal: while some still fail to provide adequate visualization and optimal access to the vascular equator, others are substantially more invasive and carry an increased risk of maternal morbidity.

Flexible endoscopy offers a promising solution by substantially facilitating exploration of the placental surface. Ahmad et al. developed a prototype FF (10.5 Fr) with a 90° deflectable tip that was validated both in a phantom model and in vivo in a pregnant ewe, where it successfully enabled visualization and coagulation of anteriorly located vascular targets [15]. In our study, we used a device with 270° tip deflection, allowing extended mapping of the vascular equator particularly at the outset of the procedure. Importantly, while the system permits up to 270° deflection for placental mapping, the introduction of a 600 μm laser fiber reduces the effective deflection to 90°. Smaller‐diameter fibers (e.g., 400 μm), which are also commonly used in laser fetoscopy, allow for greater angulation (up to 180°).

A critical feature of the device tested in our study is the straight rigid cannula, which enables accurate orientation of the scope within the uterine cavity. Unlike in urological endoscopy, where flexible devices are advanced within narrow hollow organs, fetoscopy is performed in a large fluid‐filled environment; without rigid cannula guidance, navigation and tip deflection become ineffective. We consider this element essential for clinical application in fetal surgery. The device evaluated in our study is already commercially available, which facilitates broader dissemination.

To date, the only clinical series is the Mexican study by Cruz‐Martínez et al., who reported outcomes with a similar flexible video fetoscope in 21 pregnancies with anterior placenta, without the use of a rigid cannula [5]. Among the seven cases in which the equator was inaccessible with a conventional curved scope, six (85.7%) required additional laser coagulation due to either persistent vascular patency or residual anastomoses despite initial treatment. These findings highlight the potential of flexible endoscopy to complete otherwise incomplete procedures. In our study, we confirmed the feasibility of flexible fetoscopy in a simulated TTTS scenario. Although the Solomonization path was more distant from the vascular equator, it was consistently located in a more anterior position and less frequently crossed the intertwin membrane. This pattern likely reflects the increased deflection capacity of the device, which enabled broader exploration of the placental surface and allowed operators to adopt an anterior trajectory, avoiding transmembrane coagulation. However, this shift in trajectory resulted in a greater deviation from the theoretical vascular equator, highlighting a trade‐off between membrane preservation and spatial accuracy of coagulation.

A key strength of our study lies in the recruitment of an expert population of fetal surgeons participating in a national reference meeting, representing a large proportion of active fetoscopy operators. This ensured that the device was evaluated by experienced users in conditions close to real clinical practice. To minimize the risk of memorization bias from repeated exposure to the simulator, we deliberately analyzed only the performance with the first device used by each operator. This approach avoided overestimating performance with the second device, which would inevitably benefit from prior familiarization with the simulator environment.

Several limitations should be acknowledged. First, the pre‐positioning of the trocar standardized the experiment but does not reflect the individualized trocar placement used in clinical practice. This is an inherent constraint of the simulator design and may influence which instrument appears more advantageous. Second, the assessment of vessel approach (tangential vs. perpendicular) and the delineation of Solomonization paths were based on visual evaluation without objective measurements, which may introduce some degree of interpretation bias. However, this limitation was mitigated by the joint evaluation of two observers, ensuring a standardized assessment. In addition, the comparison was performed using a straight rigid fetoscope, whereas curved rigid fetoscopes are often used in clinical practice for anterior placentation. However, the experimental configuration was designed such that the placental equator remained accessible with both devices, allowing a standardized comparison of performance while isolating the effect of instrument flexibility. Importantly, the primary objective was not to demonstrate the superiority of flexible over rigid instrumentation but to assess the feasibility and operator perception. Further studies are warranted in models with strictly anterior placentation to compare surgical performance using flexible versus curved rigid fetoscopes.

Participants consistently stressed the need for additional training before clinical implementation. This requirement is highly relevant and echoes the experience of urology and gastroenterology, where flexible endoscopes have long been used and where training programs have addressed the initial challenges of orientation and the risk of loop formation (“looping”) [16, 17]. Simulation‐based curricula appear particularly critical in the context of fetoscopic laser surgery, which combines high technical complexity, substantial fetal risk, and low operator exposure due to the rarity of TTTS. Therefore, our results underscore the importance of dedicated simulation training to ensure safe and effective adoption of flexible fetoscopy in clinical practice.

## 5 Conclusion

This randomized simulation‐based study demonstrates the feasibility of flexible fetoscopy for the management of TTTS with anterior placentation. The flexible fetoscope facilitated vascular access and promoted a more anterior trajectory compared with the rigid instrument. These findings highlight its potential clinical value and underscore the need for dedicated training.

## Funding

This study was supported by the DEVADAN Endowment Fund (Développement et Évaluation de l’Activité de Dépistage Anténatal et Néonatal) and the ADOL Association (Association pour le Développement de l’Obstétrique Lyonnaise). Their financial contributions made it possible to acquire the simulator used in this study.

## Ethics Statement

The study was conducted in accordance with the ethical principles of the Declaration of Helsinki and was approved by the Institutional Review Board (approval number CEROG 2025‐OBS‐0902).

## Consent

This study did not involve patients. All participants agreed to take part and provided written informed consent prior to participation.

## Conflicts of Interest

The authors declare no conflicts of interest.

## Supporting information


Supporting Information S1

**Video S1:** Video illustrating the simulator and the use of both rigid and flexible fetoscopic devices in this setting, with simultaneous internal and external views.

## Data Availability

The data that support the findings of this study are available from the corresponding author upon reasonable request.
