# Supplementary material for: Performance of a Flexible Versus Rigid Fetoscope in the Simulated Management of Twin‐to‐Twin Transfusion Syndrome
Source: Prenat Diagn. 2026 Jul 5;46(9):1472–9. doi: 10.1002/pd.70213 (PMC13446528; doi:10.1002/pd.70213)
Supplement: Supplementary file 1 [file PD-46-1472-s001.docx]

**Supporting Information video caption:** Video illustrating the simulator and the use of both rigid and flexible fetoscopic devices in this setting, with simultaneous internal and external views. The flexibility of the flexible fetoscope is demonstrated without a laser fiber in the working channel, and with 400 µm and 600 µm laser fibers in place.
